# Genome-based engineering of ligninolytic enzymes in fungi

**DOI:** 10.1186/s12934-021-01510-9

**Published:** 2021-01-21

**Authors:** Michael Dare Asemoloye, Mario Andrea Marchisio, Vijai Kumar Gupta, Lorenzo Pecoraro

**Affiliations:** 1 School of Pharmaceutical Science and Technology, Tianjin University, Nankai District, 92 Weijin Road, Tianjin, 300072 China; 2 Biorefining and Advanced Materials Research Center, Scotland’s Rural College (SRUC), Kings Buildings, West Mains Road, Edinburgh, EH9 3JG UK

**Keywords:** Biosynthetic pathways, CRISPR-cas, Fungi, Fungal secretome, Gene editing, Heterologous protein expression, Ligninolytic enzymes, Synthetic promoters, Transcription activation

## Abstract

**Background:**

Many fungi grow as saprobic organisms and obtain nutrients from a wide range of dead organic materials. Among saprobes, fungal species that grow on wood or in polluted environments have evolved prolific mechanisms for the production of degrading compounds, such as ligninolytic enzymes. These enzymes include arrays of intense redox-potential oxidoreductase, such as laccase, catalase, and peroxidases. The ability to produce ligninolytic enzymes makes a variety of fungal species suitable for application in many industries, including the production of biofuels and antibiotics, bioremediation, and biomedical application as biosensors. However, fungal ligninolytic enzymes are produced naturally in small quantities that may not meet the industrial or market demands. Over the last decade, combined synthetic biology and computational designs have yielded significant results in enhancing the synthesis of natural compounds in fungi.

**Main body of the abstract:**

In this review, we gave insights into different protein engineering methods, including rational, semi-rational, and directed evolution approaches that have been employed to enhance the production of some important ligninolytic enzymes in fungi. We described the role of metabolic pathway engineering to optimize the synthesis of chemical compounds of interest in various fields. We highlighted synthetic biology novel techniques for biosynthetic gene cluster (BGC) activation *in fungo* and heterologous reconstruction of BGC in microbial cells. We also discussed in detail some recombinant ligninolytic enzymes that have been successfully enhanced and expressed in different heterologous hosts. Finally, we described recent advance in CRISPR (Clustered Regularly Interspaced Short Palindromic Repeats)-Cas (CRISPR associated) protein systems as the most promising biotechnology for large-scale production of ligninolytic enzymes.

**Short conclusion:**

Aggregation, expression, and regulation of ligninolytic enzymes in fungi require very complex procedures with many interfering factors. Synthetic and computational biology strategies, as explained in this review, are powerful tools that can be combined to solve these puzzles. These integrated strategies can lead to the production of enzymes with special abilities, such as wide substrate specifications, thermo-stability, tolerance to long time storage, and stability in different substrate conditions, such as pH and nutrients.

## Background

Ligninolytic enzymes (LEs)—also referred to as lignolytic enzymes or lignin modifying enzymes (LMEs)—are proteins that catalyze modifications or degradation of lignin into less complex molecules, to produce CO_2_ eventually [1, 2]. Many fungal species produce LEs and possess complex enzyme systems for lignin degradation, including laccase (LCC), lignin peroxidase (LiP), manganese peroxidase (MnP), and versatile peroxidase (Vp). These enzymes are characterised by broad specificity in degradation/mineralization of different complex substrates such as wood, paper, animal feeds, pesticides, biofuels, and hydrocarbons [2–6]. The importance of LEs in biotechnological, industrial, and environmental applications—especially in the production of bioenergy or biomaterials, is worldwide recognized [7]. However, many natural products (NPs) are not naturally produced in enough quantities for human or industrial use. Beside NPs, specific fungal enzymes, which either play a role in metabolic pathways or are secreted to carry out their activities in the environment, have grabbed the attention of the Synthetic Biology community.

Metabolic pathway engineering, therefore, has become quickly an important branch of Synthetic Biology, whose goal is to optimize the synthesis of chemical compounds of interest in various fields, from pharmaceutics to biofuel [8]. For instance, the production in *Saccharomyces cerevisiae* (Desm.) Meyen of the artemisinic acid, a precursor of the antimalarial drug artemisinin—usually extracted from the plant *Artemisia annua* L.—is still one of the most celebrated successes in Synthetic Biology [9]. Beside plants, filamentous fungi are another primary source of natural products (NPs), which are beneficial to human health. Genes that drive the expression of NPs are, generally, located into small regions of the fungal genome, giving rise to so-called biosynthetic gene clusters (BGCs).

As an engineering science, Synthetic Biology aims at the rational design and model-driven in vivo implementation of biological systems that carry out specific tasks [10]. The first Synthetic Biology artifacts were genetic circuits, more precisely transcriptional networks, able to mimic electronic functions such as oscillations, toggle switches, and Boolean gates [11]. Circuits, initially, were hosted by bacteria—often *Escherichia coli*—and required the construction of a handful of transcription units i.e. DNA sequences where a promoter is followed, in the order, by a ribosome binding site (RBS), a coding sequence (CDS), and finally, a terminator. In parallel, computational tools were developed to facilitate the design, modeling, and analysis of genetic circuits [12, 13]. The increasing complexity of synthetic gene circuits led to faster and more reliable techniques for DNA assembly, such as the Golden Gate [14] and the isothermal assembly [15] method, where multiple DNA sequences are joined in a single experiment. Signaling and metabolic pathway rewiring and reconstruction in heterologous hosts were among the first Synthetic Biology achievements in eukaryotic cells [16].

In bioinformatics, genome mining refers to the identification of features of an organism through the analysis of its genome. In this context, software has been written ad hoc for the analysis of fungal genomes in order to both trace BGCs associated with known NPs and discover silent BGCs that, once activated, potentially lead to the expression of novel, valuable compounds [17, 18]. Algorithms for the identification of gene clusters into unannotated or partially annotated genomes are very accurate but can detect only BGCs associated with well-defined families of NPs, such as polyketides and RiPPS (ribosomally synthesized and post-translationally modified peptides). Secondary metabolite unique regions finder (SMURF) [19], antibiotics and secondary metabolites analysis shell (antiSMASH) [20, 21], secondary metabolite by inter-pro-scan (SMIPS), and cluster assignment by islands of sites (CASSIS) [22] are popular software for secondary metabolite detection and BGC recognition. More obscure, silent fungal BGCs have been discovered by means of different computational tools. For instance, motif-independent de novo detection algorithm for secondary metabolite biosynthetic gene clusters (MIDDAS-M) [23] has brought to light the BGC of ustiloxin B and MIPS-GS [24] has permitted to decipher the kojic acid BGC in *Aspergillus flavus* var. *oryzae* (Ahlb.) Kurtzman, M.J. Smiley, Robnett & Wicklow. These computational works, combined with experimental evidence, have laid the foundation of the MIBiG (Minimum Information about a Biosynthetic Gene cluster) [25] repository and data standard. MIBiG offers a broad collection of building blocks (genes, enzymes, and protein domains) for the in silico design of novel biosynthetic pathways [26, 27].

Synthetic Biology has also put forward novel techniques for BGC activation *in fungo* and heterologous reconstruction of BGC in microbial cells [28]. Control of single genes or entire pathways *in fungo* has been achieved through the expression of transcription factors based on bacterial proteins, a technique already largely exploited in mammalian and yeast cells. The Tet-ON system [29] has allowed the synthesis of a novel NP—named fumipyrrole—in *Aspergillus fumigatus* Fresen [30]. More recently, Rantasalo and co-authors [31] have shown that a synthetic activator, made by the fusion of the DNA-binding bacterial protein Bm3R1 [32] and the viral activation domain VP16 [33], enhances red fluorescence expression in *Aspergillus niger* Tiegh. and *Trichoderma reesei* E.G. Simmons.

Genome-based engineering of LEs in fungi is necessary in order to increase their production to meet human demands and industrial standards. To this aim, several approaches such as rational, semi-rational or directed evolution have been employed. However, Synthetic Biology methods and computational tools have not been fully exploited yet for enhancing the production of LEs in fungi or optimizing their synthesis in microbes, such as *S. cerevisiae* and *E. coli*. CRISPR (clustered regularly interspaced short palindromic repeats)-Cas (CRISPR associated) protein systems [34] seem the most promising biotechnology for large-scale production of LEs. They have already proved their efficiency in the editing, discovery, and activation of fungal genes and BGCs [35]. Moreover, on the computational side, numerous new algorithms have been written both to optimize CRISPR-Cas systems that edit specific loci in a genome and estimate potential off-target effects, which can lead even to cell death [36].

In this review, after introducing different groups of LEs together with their oxidative mechanisms, we described how to engineer LEs with improved activity, and illustrated general Synthetic Biology techniques both to build synthetic promoters and engineer new proteins (nucleases and transcription factors) for DNA editing and control of gene expression. We showed recent applications of the latter techniques to filamentous fungi and their usage in yeast to enhance the production of LEs. Finally, we discussed how Synthetic Biology methods may improve the use of LEs in biotechnology.

## Lignin modifying fungi and ligninolytic consortium

### Lignin

**Fig. 1:**
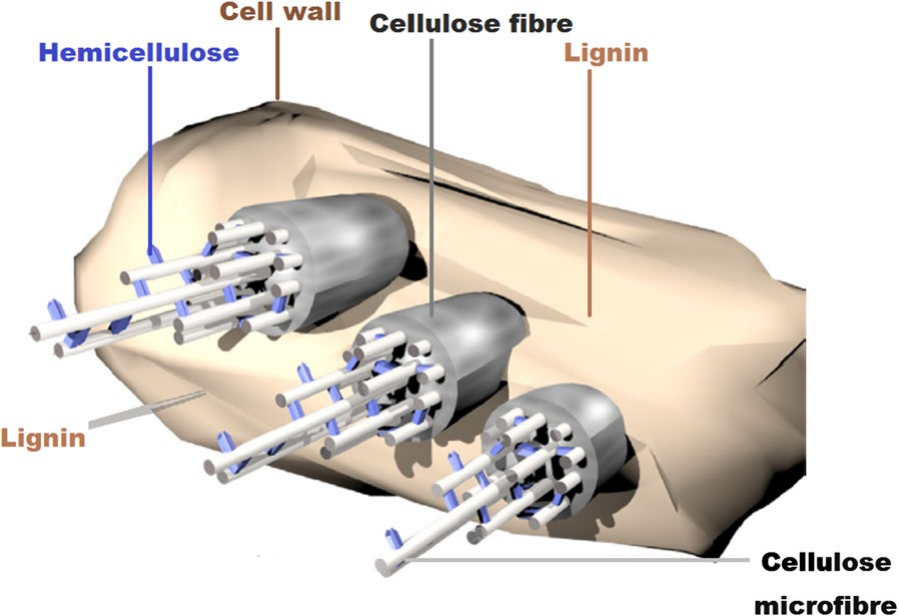
Structural sketch of lignin strengthening cellulose and hemicellulose biomass in the cell wall. Figure adapted from Alcalde [87]

Lignin is one of the major structures that strengthen the cellulose and hemicellulose fibres in plant cell wall [37–39], as shown in Fig. 1. This peculiar molecule plays an essential role in plant physiology by enhancing the transportation of water and helping plants to resist the attack of pathogens [40]. It is a complex aromatic polymer constituted by heterogenous components of mono-, di-, and non-methoxylated phenylpropaniol moieties with non-hydrolysable linkages between the subunits of p-hydroxycinnamyl alcohols (i.e., the coniferyl, coumaryl and sinapyl alcohols) [41, 42]. Cellulose as an organic polysaccharide polymer consists of a chain of many hundreds or thousands of β-glucose 1–4 cross-linked with D-glucose units. It is an important structural components of plant’s primary cell wall and is also abundant in the cell walls of algae and the fungal class of oomycetes. Moreover, it has been discovered that some bacteria can secret cellulose biomers to form biofilms [39]. Cellulose appears as a crystalline white structure, has a melting point of 260–270 °C with 1.50 g/cm^3^ density and 162.1406 g/mol molar mass, makes 90% of cotton fibre, about 57% in hemp, and constitutes 40–50% of wood [39]. Hemicellulose, on the other hand, exists as an heteropolymer of organic polysaccharide like arabinoxylans. It usually appears along with cellulose in plant cell wall. Unlike cellulose, hemicellulose is an amorphous structure less strong or resistant to hydrolysis.

Lignin and cellulose components of plant cell represent the most abundant natural biomass on earth (lignocellulose biomass). The lignin portion of plant biomass is estimated to be produced at a rate of about 200 billion tons/year and it is responsible for larger quantities of carbon fixed in terrestrial ecosystems [42, 43]. Lignin is found in large quantities in woody plants, accounting for about 26–32% dry weight in soft woods and 20–25% in hard woods, and it is estimated to constitute about 15–21% of dry mass in corn stover, and 5–17% in wheat straw [42]. Large chunk of dead lignocellulose components in plant/wood materials are decomposed by microorganisms leading to a fundamental carbon recycling in the biosphere. Although lignin is difficult to degrade for most of microorganisms, due to its complex nature, some fungi have the capability to decay this molecule by producing specific enzymes, which have been studied intensely [43, 44]. Among lignin-decaying fungi, the white-rot basidiomycetes are known to be particularly efficient due to their secretion of vast oxidoreductase enzymes [45].

### Lignin modifying fungi

Fungi are more efficient in lignin degradation than other microbes [42]. Wood-decaying basidiomycetes have been divided into two groups, white- and brown-rot basidiomycetes, based on their morphological growth on woods, which reflects their different wood degradation activity. White-rot fungi (WRF) preferentially grow on deciduous trees and gradually degrade cellulose and lignin using high redox-potential oxidoreductive enzymes commonly referred to as LEs. This degradation mechanism results in the typical white colour of the cellulose-enriched wood left behind [46]. On the contrary, brown-rot fungi mostly colonize conifers and primarily degrade hemicellulose and cellulose, leaving a brown coloured residual wood enriched in the darker lignin [47]. However, both white-rot fungi and some brown-rot fungal species, such as *Gloeophyllum trabeum* (Bourdot & Galzin) Domański, Orloś & Skirg [48], are particularly predisposed to lignin degradation/mineralization due to their production of several oxidoreductase enzymes for conversion of lignin to H_2_O and CO_2_ [48–50]. Examples of common wood decaying white-rot fungi are *Phanerochaete chrysosporium* Burds., *Armillaria* sp.*, Phlebia* sp.*, Phellinus pini* (Brot.) Pilát*, Schizophyllum commune* Fr.*, Dichomitus squalens* (P. Karst.) D.A. Reid*, Trametes versicolor* (L.) Lloyd*, Daldinia concentrica* (Bolton) Ces. & De Not., while the brown-rot fungi include *Laetiporus portentosus* (Berk.) Rajchenb., *Monilinia fructicola* (G. Winter) Honey*, Fomitopsis lilacinogilva* (Berk.) J.E. Wright & J.R. Deschamps*,* and *Postia placenta* (Fr.) M.J. Larsen & Lombard.

Production of LEs has not yet been reported in Chytridiomycota [51], whereas reports are increasing from the Ascomycota (mould group) and Mucoromycota [52]. The soft rot fungi (ascomycetes) such as *Xylaria, Fusarium, Penicillium, Trichoderma,* and *Aspergillus* species can degrade the softer section of wood that is constituted by a lower proportion of lignin [53]. These fungi have been described to be capable of mineralizing lignin and have shown wood rotting activities, although in lesser rate than in basidiomycetes [54–57]. In addition, some fungal plant pathogens have been associated with lignin-degrading activities. For instance, extracellular laccase production was found in *Botrytis cinerea* Pers. a causal agent of soft rot disease in *Daucus carota* L.*, Greeneria uvicola* (Berk. & M.A. Curtis) Punith, the well-known rot pathogen of wine grape (*Vitis vinifera* L.), and many others such as *Pythium* and *Fusarium* species [58].

### Ligninolytic enzyme consortium

**Fig. 2:**
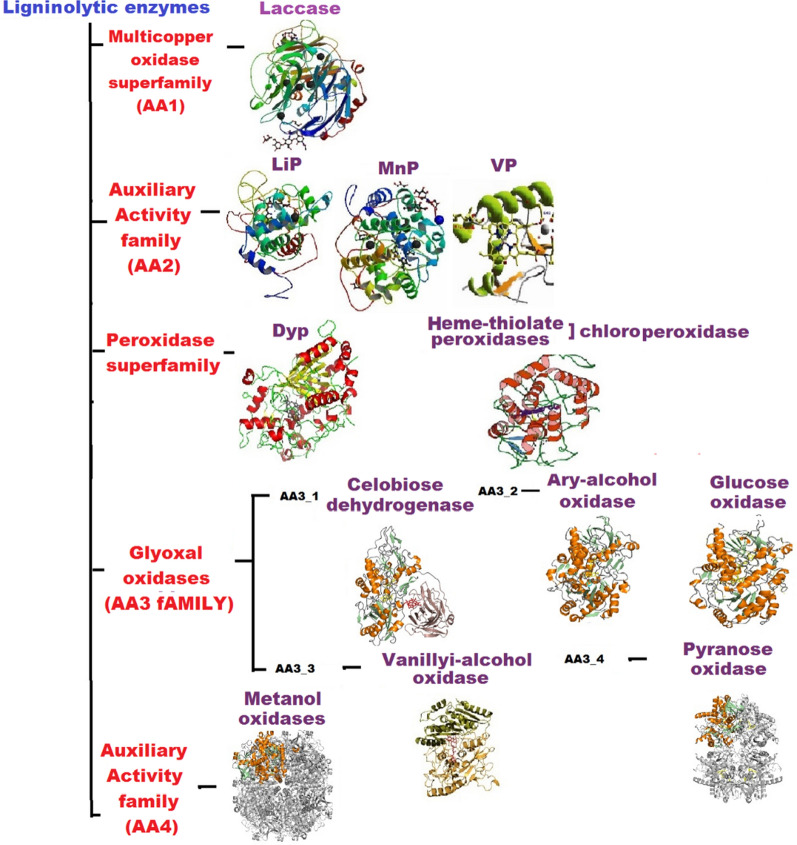
Classification and structure of common fungal ligninolytic enzymes. LiP = Lignin peroxidase, MnP = Manganese peroxidase, VP = Versatile peroxidase, Dyp = Dye-decolorizing peroxidase, AA = Auxiliary activity family

Fungi efficiently degrade lignin thanks to their complex ligninolytic bio-refineries with ability to produce different cassettes of ligninolytic secretome (TLS). These are mostly constituted by enzymes known to act as natural oxidative systems (Table 1; Additional file 1: Table S1), which catalyze non-specific reactions leading to depolymerization/degradation of lignin [59]. Many of them are catalogued in Carbohydrate-Active enZymes Database (CAZy) as lignin-degrading auxiliary enzymes (LDA) or commonly as auxiliary activity (AA) enzymes (CAZy; http://www.cazy.org/). The most common LEs in fungi (Fig. 2) are grouped in different families that can be found in https://www.cazypedia.org/:

i. Multicopper oxidase superfamily (AA1), such as laccase (Lcc).
ii. Auxiliary Activity family (AA2), which are peroxidases, including lignin peroxidase (LiP), manganese peroxidase (MnP), and versatile peroxidase (VP).
iii. Peroxidase superfamily, such as dye decolorizing peroxidases (DyP) and heme-thiolate peroxidases (HTPs).
iv. Auxiliary Activity family (AA3), which are glyoxal oxidases (GLOX);
v. Subfamily AA3_1; for example, cellobiose dehydrogenase.
vi. Subfamily AA3_2; for instance, aryl-alcohol oxidase/dehydrogenase, galactose oxidase, glucose dehydrogenase, glucose oxidase, and pyranose dehydrogenase.
vii. Subfamily AA3_3, including alcohol oxidases, such as vanillyl alcohol oxidase and benzoquinone reductase.
viii. Subfamily AA3_4, including pyranose oxidase.
ix. Auxiliary Activity family (AA4), which are vanillyl-alcohol oxidases.

**Table 1:** Family of ligninolytic enzyme consortium reported in fungi

Fungal Enzyme	Name of CAZy/Systematic name/EC no	Fungi producing the enzyme
Lignin peroxidase (LiP)	AA2/ (3,4-Dimethoxyphenyl)methanol or hydrogenperoxide oxidoreductase/EC1.11.1.14	Produced by some white rot fungi like *Phanerochaete chrysosporium, Phlebia radiata,* and *Trametes versicolor*
Manganese peroxidase (MnP)	AA2/ hydrogen-peroxide oxidoreductase/EC1.11.1.13	Produced by white rot wood decomposing mushrooms and litter-decomposing fungi such as *Dichomitus squalens, Agaricus bisporus* and *Agrocybe praecox*
Laccase (LCC)	AA1_1/Benzenediol oxygen oxidoreductase or 4 Benzenediol/EC1.10.3.2	Produced by ascomycetes and basidiomycetes fungi
Versatile peroxidase (VP)	AA2/hydrogen-peroxide oxidoreductase/EC1.11.1.16	Only known in few white rot species such as *Pleurotus ostreatus* and *Bjerkandera adusta*
Glyoxal oxidase (GLX)	AA5_1/NA/ EC1.1.3	White rot and brown rot basidiomycete species (e.g., *P. chrysosporium*)
Aryl-alcohol oxidase (AAO)	AA3_2/Aryl-alcohol:oxygen oxidoreductase/EC1.1.3.7	Produced in white rot fungi such as *Pleurotus eryngii*
Dye-decolorizing peroxidase (DyP)	NA/hydrogen-peroxide oxidoreductase/EC1.11.1.19	Common in many ascomycete, white rot and litter-decomposing basidiomycetes such as *Geotrichum candidum, B. adusta, Auricularia auricula-judae, Aspergillus* sp.
Chloroperoxidase (CPO)	NA/Chloride:hydrogen-peroxide oxidoreductase/EC1.11.1.10	Produced in some litter-decomposing basidiomycetes such as *Agaricus bisporus*
Unspecific peroxygenase (UPO)	NA/Substrate:hydrogen peroxide oxidoreductase (RH-hydroxylating or -epoxidizing)/EC1.11.2.1	Produced in some litter-decomposing basidiomycetes such as *Agrocybe aegerita*
Cellobiose hydrogenase (CDH)	AA3_1/Cellobiose:acceptor 1-oxidoreductase/EC1.1.99.18	Produced in many ascomycetes and basidiomycetes such as *Myceliophtora thermophila*, and *D. squalens*
Glucose oxidase (GOX)	AA3_2/β-D-Glucose:oxygen 1-oxidoreductase/EC1.1.3.4	ascomycetes and basidiomycetes (e.g., *Aspergillus niger, P. chrysosporium*)
Methanol oxidase (MOX)	AA3_3/Alcohol:oxygen oxidoreductase/EC1.1.3.13	Produced by some ascomycetes and basidiomycetes (e.g., *Penicillium amagasakiense, P. chrysosporium, Gloeophyllum trabeum*)
Pyranose 2-oxidase (P2O)	AA3_4/Pyranose:oxygen 2-oxidoreductase/EC1.1.3.10	Produced by some basidiomycetes (e.g., *P. chrysosporium, Trametes ochracea*)
Vanillyl-alcohol oxidase (VAO)	AA3_3/Vanillyl alcohol:oxygen oxidoreductase/EC1.1.3.38	Produced by some ascomycetes, *Penicillium simplicissimum*
Galactose oxidase (GAO)	AA3_2/D-Galactose:oxygen 6-oxidoreductase/EC1.1.3.9	Produced by some ascomycetes (e.g., *Fusarium graminearum, Aspergillus nidulans*)
Benzoquinone reductase (BQR)	AA6/NADPH:p-benzoquinone oxidoreductase/EC1.6.5.6	Produced by some white and brown rot basidiomycetes (*P. chrysosporium, G. trabeum)*

*NA* not available

Genes that encode the expression of these enzymes have been widely reported in the genomes of many WRF [60–62]. LiP, MnP, and VP are common LEC of class II heme-containing peroxidases [63], which are grouped in AA1-AA3 enzyme family due to their ability to catalyse the oxidation of hydrogen peroxide as co-substrate. These TLS are also very much involved in high-redox catalytic oxidation of complex aromatic hydrocarbons (Additional file 1: Table S2). Peroxidases (LiP, MnP, and VP) generally have a conserved primary, secondary and tertiary architectures including Ca ion binding sites, heme-pocket residues, and four disulfide bridges [64].

The first discovered enzyme from the group of LEs is the LiP, which was independently discovered in 1983 by Tien and Kirk [1] and Glenn et al. [3], from *P. chrysosporium*. This enzyme was initially named ligninase, but was later reported in some WRF belonging to *Trametes, Phanerochaete, Phlebia*, and *Bjerkandera* genera under the name of lignin peroxidase [62]. LiP was found to be able to speed up lignin degradation and oxidation of complex hydrocarbons through a vast electron transfer to a heme cofactor [65]. For example, LiP H8 was found to exhibit catalytic reaction in the presence of redox active substrate containing tryptophan in *P. chrysosporium* [66]. Similar observation was later made for VP by Ruiz-Dueñas et al. [67]. Both VP and LiP differ from other LEs because of their redox potentials that are estimated to range between 1.4 and 1.5 V during direct oxidation of non-phenolic lignin. These peculiar redox potentials allow VP and LiP to oxidize long chain polycyclic aromatic hydrocarbons through Cα-Cβ ring cleavage and cleave dimeric lignin compound [68]. A key example of LiP action is the oxidation of the aromatic substrate veratryl alcohol (3,4-dimethoxybenzyl alcohol) to veratraldehyde, while VP acts directly on aromatic compounds with Mn oxidizing efficiency, which makes the activity of this enzyme similar to both LiP and MnP [42]. The latter is another example of LE that is commonly produced by wood- and litter-decomposing basidiomycete fungi and has been found involved in catalytic oxidation of phenolic substrates through the oxidation of Mn^2+^ to Mn^3+^ [28].

MnP is a LE that has manganese binding sites constituted by three acidic amino acid residues that coordinate Mn^2+^ (i.e. Glu-35, Glu-39, and Asp-179), as revealed in *P. chrysosporium* [28, 69]. Chelation of manganese iron Mn^3+^ by dicarboxylic acid is generally observed in reactions that involve MnPs. A typical example of such reaction is represented by the oxidation of oxalic acid into more stable form. In lignin degradation, chelated Mn^3+^ is diffused into cell wall lignin through the micropores, which are less accessible for larger enzymes [28, 69]. For MnP it may not be as easy as for LiP and VP to oxidize non-phenolic lignin, due to its lower redox potential, which was estimated around 1.0–1.2 V [68]. However, its action on non-phenolic lignin is generally mediated by MnP-lipid systems of lipid peroxidation, which generates reactive intermediates of peroxyl radicals. Recently, genetic models that encode typical manganese and versatile peroxidases, based on conserved Mn-binding sites and tryptophan as mediator, were reported [60]. In these models, new superfamilies of fungal peroxidases i.e. dye decolorizing peroxidases (DyPs) and HTPs were described. These enzymes, which are not phylogenetically related to LiPs, MnPs or VPs and are also commonly associated with lignin modification/degradation, have been reported in many fungal, bacterial and archaea genomes [70].

Laccase belongs to the group of multicopper oxidase superfamily of LEs and is listed as AA1_1 in the CAZy family [71]. This enzyme is characterized by the presence of four copper atoms and has the potential of catalyzing four different electron transfer reactions via different phenolic compounds, aromatic amines, and dye molecules with 3-dimentional structure and four substrate binding loops as well as four conserved signature sequences, which have amino acids as copper ligands. Laccase has a redox potential ranging from 0.5 to 1.0 V, and usually requires suitable mediator, such as 1-hydroxybenzotriazole and 2,2-azinobis 3-ethylbenz-thiazoline-6-sulfonate (ABTS). This ligninolytic enzyme has a wide range of substrate polymers, from non-phenolics to larger organics. Generally, the laccase-like multicopper oxidase (LMCO) family is phylogenetically divided into sensu*-stricto* laccases and iron oxidizing Fet^3^-like ferroxidases, which are very common in formation of pigments and ascorbate oxidases [72]. The LMCO family has been generally associated with morphogenesis and pathogenesis in plants [73].

DyP is a very useful enzyme due to its high redox potentials (1.2–1.5 V), which were found to enable it to oxidize different types of dyes, such as phenolics and carotenoids [74, 75], like other peroxidases. DyP was also found to be able to oxidize veratryl alcohol and non-phenolic lignin at low-level [76]. The first DyP with ability to oxidize Mn^2+^ was detected in *Pleurotus ostreatus* (Jacq.) P. Kumm. [77, 78], but the biological role of this enzyme is still not clearly understood. Besides, there have been similar reports on some groups of LEs called nonspecific peroxygenases (UPOs) in different basidiomycetes genera, such as *Agrocybe, Marasmius*, and *Coprinellus* [79]. These enzymes belong to the superfamily of versatile HTPs that catalyze the oxidation of aromatic and aliphatic hydrocarbons [80, 81]. UPOs have also been reported with potentials to catalyze the degradation of non-phenolic β-O-4 lignin dimers [68, 82], although their physiological roles have not been described.

Several other fungal enzymes that act as oxidases, according to enzyme commission classification (ECC), have been found associated with lignin and wood degradation, often leading to the evolution of H_2_O_2_, as for many LEs, like Mn (III)-mediated oxidation of some organic acids that are secreted by fungi [83]. Glucose-methanol-choline oxidoreductases are examples of oxidase enzymes which are classified in superfamily AA3. These enzymes, in nature, are flavoproteins with flavin-adenine dinucleotide-binding domain and two conserved histidines [84, 85]. Enzymes in superfamily AA3, including cellobiose dehydrogenase (CDH), glucose oxidase (GOX), aryl oxidase (AAO), methanol oxidase, pyranose-2 oxidase (P2O), etc. (Table 1) share similar structures as shown in Fig. 2 and act in similar way, for instance by oxidation of alcohol moiety to aldehydes. Another example of fungal oxidases is represented by the copper radical super-family, which includes glyoxal oxidases (GLOX). These are copper metallo-enzymes characterized by high specificity for oxidation of simple aldehydes into carboxylic acids. GLOX have unusual active site free radical-couple copper similar to those of galactose oxidases. Given their high specificity for aldehydes, GLOX have attracted the interest of researchers to a larger extent compared to other members of copper radical oxidases, and they have also been physiologically linked to LiPs in the fungal species *P. chrysosporium*, for the conversion of H_2_O_2_ in lignin degradation [86].

Fungal LEC has been widely investigated; according to Alcalde [87], the US Department of Energy invested funds in research projects for sequencing of more than 80 fungal genomes, with the aim to identify useful microorganisms for biorefineries of lignocellulosic biomass [88]. These enzymes are involved in a wide range of industrial applications, including the production of renewable organic compounds, such as fermented products, antibiotics, and biopolymers. LEs from fungi can also be very useful for the production of biofuels (bioethanol and biobutanol) and biofuel cells, for biomedical applications (biosensors), bioremediation, and in pulp and textile industries. However, these enzymes are produced naturally in small quantities that may not meet the industrial or market demands. Therefore, there is need for biotechnological enhancement of LEs production in fungi. Recent advancements in computational and synthetic biology systems provide the possibility to manipulate fungal BPs for production of LEs with desired properties in terms of reactivity, specificity, and catalytic potentials.

## Engineering new ligninolytic enzymes

**Fig. 3:**
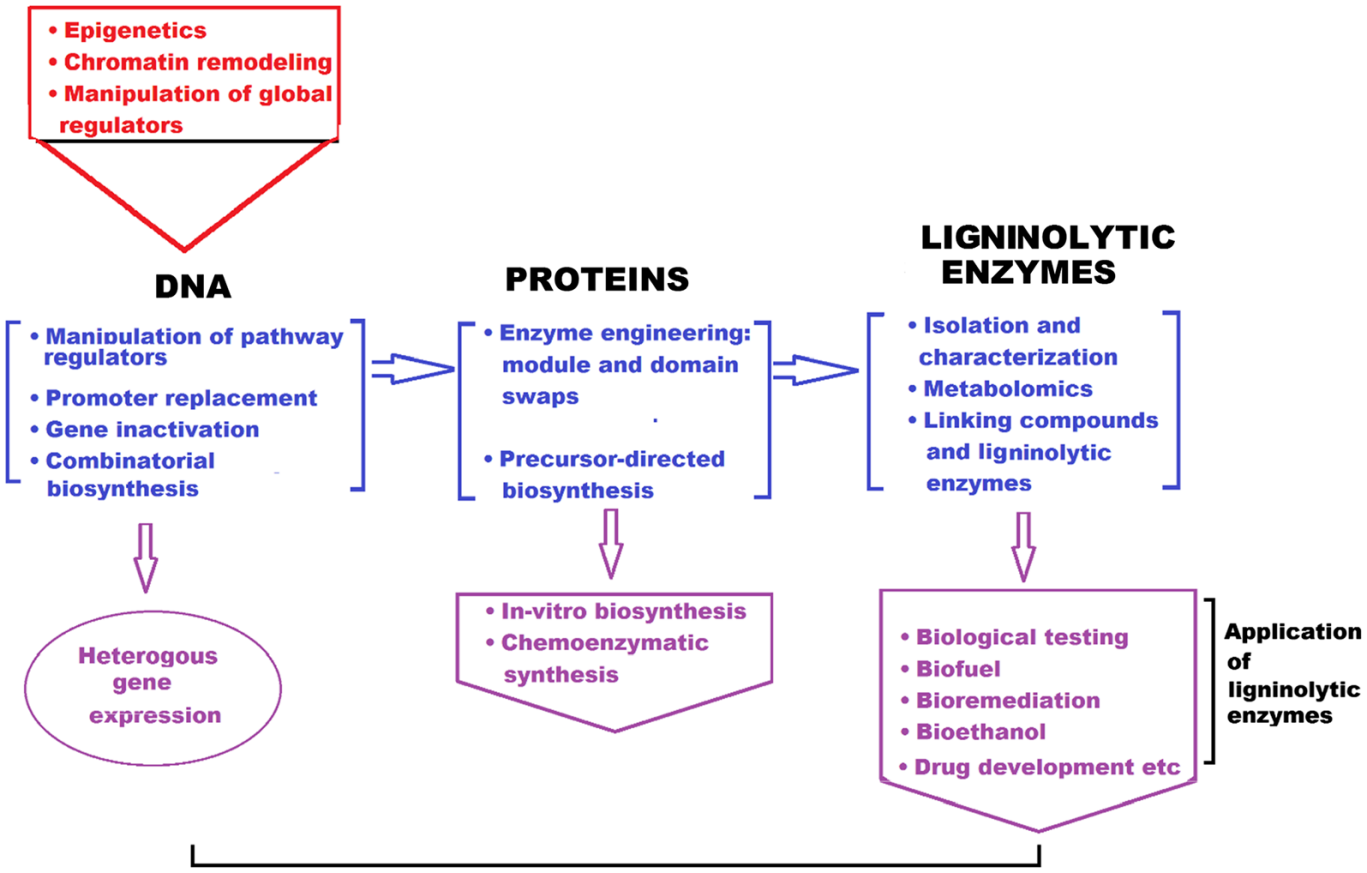
Identification and screening of ligninolytic enzymes in postgenomic era

The number of already described fungal species was recently estimated in 120,000 out of a total of 2.2–3.8 millions [89]. More than 1000 fungal genomes have been sequenced and annotated. They are available at the Department of Energy Joint Genome Institute [90], the Broad Institute Fungal Genome Initiative (https://www.broadinstitute.org/fungal-genome-initiative), and the FungiDB [91, 92]. The number of genes encoding for NPs, LEs, and other secreted chemicals/enzymes strongly depends on the fungal strain and is highly variable among different genomes, as reported in Wang et al. [93]. Libraries of new ligninolytic enzymes (Fig. 3) are obtained via three main protein engineering methods: (1) Directed evolution (DE) (2) Rational and (3) Semi-rational approaches.

The DE involves either random recombination of a set of similar sequences (such as gene shuffling) or the introduction of random changes in a single protein sequences, e.g. via error-prone PCR, to engineer improved proteins. The structural information of an enzyme is not necessarily required for DE and variations can occur, at random positions, far from the active site. These changes are often small and can require several rounds of evolution to screen a high number of variants. In fungi, DE has led to the production of LEs characterized by novel features such as tolerance to extreme pH and a prolonged storage time [94, 95]. Moreover, several computational methods such as molecular dynamics, the Monte Carlo algorithm, and ab initio calculations facilitate the study of the interactions between enzymes and ligands/substrates, which helps DE considerably through the identification of residues, and whole regions, as targets for mutagenesis and recombination [96]. Such a computer-driven directed evolution led to the synthesis of laccases with enhanced activity from *Pleurotus ostreatus* [97, 98]. Moreover, Ouyant and Zhao [99] reported improved laccase activity from the yeast mutant 5E29 with two amino acid substitutions. Directed evolution proved to be a successful technique also for the production of laccases from non-native hosts. Bulter and co-authors [100] expressed, in *S. cerevisiae*, a laccase from *Myceliophthora thermophila* (Apinis) Oorschot and improved its catalytic efficiency via 10 DR iterations. Interestingly, the laccase gene that was introduced into the yeast cells included a cleavage site at the C-terminal tail that favoured the protein biosynthesis in the Golgi compartment for packing of mutant into vesicles. Later, Zumárraga et al*.* [101] further improved this evolved variant through synergistic saturation mutagenesis, which highlighted the importance of the C terminus in ascomycete laccases.

Directed evolution methods have also been adopted for the enhancement of fungal peroxidases/peroxygenases. VP an LE from an oyster mushroom *Pleurotus eryngii* (DC.) Quel. were successfully expressed with enhanced activities and thermostability in *S. cerevisiae* by Morawski et al. [102] and Garcia et al. [103]. This enzyme was later was evolved for better oxidative stabilization by Gonzalez-Perez et al. [104]. Directed evolution was also demonstrated by Miyazaki-Imamura et al. [105] to enhance the stability of MnP from a basidiomycete fungus *P. chrysosporium* in the presence of hydrogen peroxide via an in vitro expression system. Ryu et al. [106, 107] demonstrated enhanced production of LiP from this fungus by yeast surface display. In another study carried out by Molina-Espejaet al. [108], a specific UPO from *Agrocybe aegerita* (V. Brig.) Singer was successfully expressed in *S. cerevisiae* via induced mutations in native signal peptides and mature protein, they also used that opportunity to develop tandem yeast-expression system that enables UPO production in *P. pastoris*. In addition, heterologous expression of CDH from *Myriococcum thermophilum* (Fergus) Aa. was demonstrated in *S. cerevisiae* by Sygmund et al. [109]. They however observed best CDH variant in *Pichia pastoris* (Guillierm.) Phaff via a modification in oxidative and reductive FAD half-reaction.

Rational approach for enzyme engineering is based on the use of protein structures together with biochemical and molecular modelling data for the introduction of site-specific mutagenesis. This approach has a high probability to find beneficial mutations with a consequent reduction in library size. As such, it demands less effort and time. Among rational approaches, molecular evolution has been employed for the production of enzymes with improved catalytic activities and higher tolerance to solvents and temperature [110]. Molecular evolution pursues an enhancement of fungal enzyme activity via molecular modifications (e.g. direct site mutations) driven by structural considerations. This technique has been adopted to increase the oxidative capacity of some LEs such as laccases, which degrade non-phenolic substrates, on bulky phenolic molecules [111, 112].

Semi-rational design, on the other hand, combines the use of rational and random protein design to create smaller and smarter libraries from biochemical and/or structural data [113]. Site-saturation mutagenesis is a procedure used in semi-rational design to modify hotspot residues in an enzyme. Here, one or more amino acids are replaced, at their positions, by all other possible amino acids. CASTing (combinatorial active site saturation testing) is a particular semi-rational approach that employs information derived from structural data for the identification of amino acids in particular regions (like an active site of a protein sequence) that are then mutated randomly or via site-saturation mutagenesis [114]. A different semi-rational approach, which involves the usage of focused directed evolution methods—such as combined saturation and domain mutagenesis—has been exploited to obtain enzymes with 3- to eightfold higher catalytic efficiencies [115].

## Synthetic Biology *in fungo*: gene editing and transcription activation

Synthetic biology in fungi involves the redesigning/engineering of fungal genomes to enhance their performance or give them new abilities. This demands to construct new biological parts, devices, and circuits in fungi. Many computational tools (such as those listed in Additional file 1: Table S3) are now available for use in pathway construction/enhancement in fungi, in this way, fungi can be harnessed to tackle issues in medicine, industry, human environment, and agriculture. In principle, synthetic gene circuits shall be *orthogonal* to their host cells i.e. they shall not interfere with the molecular processes of their *chassis* (the organism into which they are placed). Wanka and co-authors [116] achieved orthogonality in *Aspergillus* species by using the bacterial Tet-OFF system [117]. Here, the chimeric activator TetR-VP16 binds its operators on the DNA only in the absence of doxycyline, a derivative of the antibiotic tetracycline. The endogenous genes targeted in this work (*racA* and *gfaA* in *A. niger*; *pabaA* in *A. fumigatus*) were first deleted from and then re-inserted into the fungal genome as parts of “expression cassettes”, where they laid downstream of a minimal promoter preceded by seven copies of the tet operator, as showed in a previous work by Belli’ and co-authors [118]. Overall, these three genes were downregulated in proportion to the concentration of doxycycline in the growth medium.

Synthetic promoters made by merging sequences from different promoters (even belonging to diverse organisms) are referred to as *hybrid* and were already used before the advent of the Synthetic Biology era—for instance, see Carver et al*.* [119]. Even though the hybrid promoters guarantee orthogonality, they demand several genomic manipulations and are limited by the small number of well-characterized transcription factor-operator pairs. Transcription Activator-like Effectors (TALEs), which were discovered in plant-pathogenic bacteria belonging to the genus *Xanthomonas*, represent a tool for assembling novel transcription factor able to bind a large number of DNA sequences and, therefore, control the activity of endogenous promoters [120]. The main feature of TALEs is their modular DNA-binding domain that is made, in natural configuration, of 13 up to 28 *repeats*, most of which containing 34 amino acids (the last repeat is an exception and is only 20 amino acids long). The amino acid at position 13 determines the nucleotide that is recognized and bound by each repeat. For this reason, it is termed as base-specific residue [121].

A synthetic DNA-binding domain is assembled by joining as many repeats as there are nucleotides in the target sequence. Each repeat, usually, carries at position 13 an Asparagine to bind a guanine; a Glycine to bind a thymine; an Isoleucine to bind an adenine; or an Aspartic Acid to bind a cytosine. As a constraint, only DNA sequences preceded by a thymine or a cytosine are bound by TALEs-based proteins. Despite their overall great potential [122], TAL effectors have been used, in fungi, mainly for gene editing in the form of Transcription activator-like effector nucleases (TALENs or TAL effector nucleases) upon fusion to FokI nuclease [123, 124].

CRISPR-Cas systems [125] have rapidly become a preferred solution to operate modifications also on fungal genomes. CRISPR-Cas proteins are a component of the immune system of bacteria and archaea [126] that triggers the degradation of invading DNAs. The CRISPR array, along the prokaryotic chromosome, is made of *spacers* (i.e. sequences from previously encountered DNA intruders) separated by repeats. Upon infection, the CRISPR array is, first, transcribed into a long pre-crRNA (pre-CRISPR RNA) molecule that is, then, processed into shorter crRNAs, each containing a spacer and a repeat (in some cases, a crRNA is bound to another short RNA sequence, termed tracrRNA—transactivating RNA). The spacer binds, by complementary base-pairing, the target DNA in the proximity of the protospacer adjacent motif (PAM)—a short hallmark that consists of 3–4 nucleotides. The repeat can be seen as a handle where the corresponding Cas protein—a nuclease—binds. Overall, the Cas:crRNA complex recognize, binds, and cuts—with a double-strand break—a foreign DNA sequence.

Among the six types of CRISPR-Cas systems so far discovered, type II and type V are the most popular in biotechnology because they require a single Cas protein to cleave the DNA: Cas9 [127] and Cas12a [128], respectively (to be precise, Cas12a belongs to the subtype A of type V). In order to carry out genomic modification, CRISPR-Cas systems are an easier solution than TALENs. CRISPR-Cas demand to express a short crRNA sequence (up to about 100 nucleotides) instead of engineering a complicated, modular DNA-binding domain. Moreover, TAL effectors require to be attached to a nuclease domain that, in contrast, is naturally present in the CRISPR associated proteins. Recently, applications of CRISPR-Cas9 to filamentous fungi have focused, mainly, on *Aspergillus*. In particular, efficient gene knockout has been reported in *A. niger* [129], *A. fumigatus* [130], and *A. flavus* var. *oryzae* [131]. The latter work described also the first CRISPR-Cas9-based gene editing in *Nodulisporium* sp. and *Sporormiella minima* (Auersw.) S.I. Ahmed & Cain.

Another interesting work in *A. fumigatus* showed how to utilize CRISPR-Cas9 to activate, in a non-productive strain of this fungus, the BGC for the synthesis of the antibiotic trypacidin [132]. Shi and co-authors [133] exploited CRISPR-Cas9 to rewire metabolic pathways in *Fusarium fujikuroi* Nirenberg and change, as a consequence, the product profile of the gibberellic acids. CRISPR-Cas9 has also been used to engineer BPs in many fungi [134]. The only work where CRISPR-Cas9 was used to improve the production of a ligninolytic enzyme, cellulase, is a multiplexed genome engineering in *Myceliophthora* by Liu and co-authors [135]. Here, enhanced cellulase production (about 5 folds) and activity (up to over 13 folds) resulted, with respect to the wild-type strain, after the deletion of four selected genes: *cre1*, *res1*, *gh1-1*, *and alp-1*. Other previous research papers on CRISPR-Cas9-based genome editing in filamentous fungi have been reviewed in [136].

CRISPR-Cas12a systems have been employed in fungal genomic manipulation only over the last two years. Also in this case, most of the experiments have been done in *Aspergillus*. The first proof of the working of LbCas12a (i.e. from *Lachnospiraceae bacterium*) in filamentous fungi—*A. nidulans* (Eidam) G. Winter and *A. niger*—was given in [137]. A later work, again in *A. nidulans*, was the first to use a nuclease-deficient version of a CRISPR associated protein—here LbCas12a again—to construct a synthetic activator and trigger gene expression *in fungo* [138]. The catalytically dead protein dLbCas12a was fused to the strong VPR synthetic activation domain [139] and tested, initially, on an exogenous gene encoding for a fluorescent protein. The successful production of a fluorescence signal led Roux and co-authors [138] to address the action of the synthetic activator to the endogenous *micA* gene, which permitted an increased synthesis of microperfuranone. Finally, dLbCas12a-VPR allowed the activation of a previously unknown BGC, whose product turned out to be dehydromicroperfuranone. Kwon and co-authors [140], in contrast, tested a different version of Cas12a (FnCas12 from *Francisella novicida* and AsCas12a from *Acidaminococcus* sp. BV3L6) together with Cas9 (the usual one, from *Streptococcus pyogenes*) on single-gene and multiplex-gene targeting in *Thermothelomyces thermophilus* (Apinis) Y. Marín, Stchigel, Guarro & Cano. Every CRISPR-Cas system proved to be active in this particular filamentous fungus with FnCas12a showing higher resilience than the other two CRISPR associated proteins.

CRISPR-Cas systems have not been used for the enhancement of LE expression in fungi yet. Probably, the main reason has been the lack of sequenced genomes since template genomes from different fungi show extensive variations and, therefore, do not allow for precise DNA targeting. However, recent progress in next-gen sequencing technology could facilitate fungal genomic engineering and Synthetic Biology in the incoming years.

### Heterologous expression of recombinant ligninolytic enzymes

Expression of recombinant proteins in hosts that can be cultivated easily has been encouraged to reduce the production cost. Ligninolytic enzymes that are produced by many basidiomycete fungi, such as *Myceliophthora thermophila, P. chrysosporium, Pleurotus* sp., *Trametes trogii* Berk.*,* and *Trametes versicolor* have been expressed in some ascomycete fungi. Record et al*.* [141] established over-expression of laccase *lac1* gene from the white rot fungus *Pycnoporus cinnabarinus* (Jacq.) P. Karst. (commonly referred to as Cinnabar polypore) in *Aspergillus niger*. The *lac1* gene was placed downstream of the strong constitutive promoter of glyceraldehyde-3-phosphate dehydrogenase, which provoked an 80-fold increase in the synthesis of the recombinant laccase. In a later study, Kiiskinen et al. [142] reported the production of *Melanocarpus albomyces* (Cooney & R. Emers.) Arx laccase *lcc* in *Trichoderma reesei* under the strong cellobiohydrolase *cbh* promoter. In the same year, Hoshida et al. [143] described the use of *A. flavus* var. *oryzae* and *S. cerevisiae* as hosts for overexpressing *Pycnoporus coccineus* (Fr.) Bondartsev & Singer laccase *lcc1*. Laccase synthesis was driven by the maltose inducible promoter *amyB* in *A. flavus* var. *oryzae*, whereas the galactose inducible *GAL10* promoter was employed in *S. cerevisiae.*

**Fig. 4:**
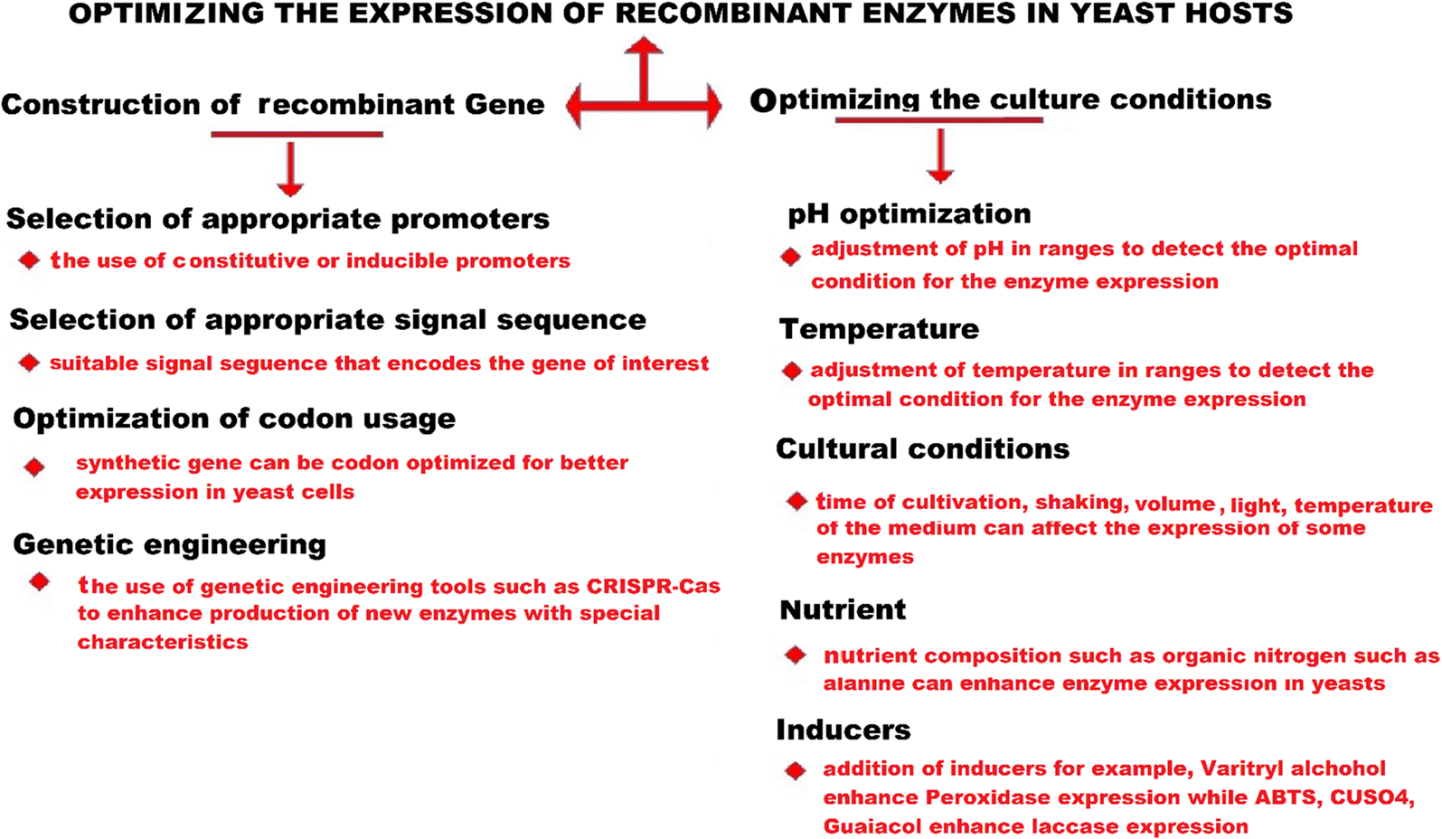
General overview on the approaches for optimizing enzyme expression in yeast

Yeasts (methylotrophs or non-methylotrophs) have post-translational modifications. Moreover, they are fast growing, have simple morphology, produce biomass, and are safe [144]. Yeasts have been established as suitable hosts for the heterologous expression of recombinant proteins [145, 146]. Examples of yeast genera commonly adopted as a chassis for the synthesis of heterologous proteins are *Saccharomyces* and *Yarrowia,* among non-methylotroph yeasts, together with *P. pastoris* and *Hansenula polymorpha* (methylotroph yeasts). Many wild-type and mutant strains of various *Saccharomyces* species (e.g. *S. cerevisiae, S. uvaruium*, and *S. boulardi*) are now available for research and industrial applications. Compared to other fungi and higher eukaryotes in general, the yeast genome is easier to modify by exploiting homologous recombination (Fig. 4).

Moreover, many constitutive and strong synthetic promoters have been characterized in several yeast species like *Kluyveromyces lactis, P. pastoris, Yarrowia lipolytica, Pichia methalonic, Cryptococcus* sp., and *S. cerevisiae* [147]. These promoters have been used to improve in yeasts the expression of different LEs from higher fungi [148]. Apart from using strong synthetic promoters, enzyme production in yeast can be enhanced by means of multicopy plasmids. These plasmids can dramatically increase the copies of a transcription unit carrying the enzyme CDS into yeast cells [145]. Episomal shuttle vectors replicate in the host cell autonomously and are commonly utilized for the expression of heterologous genes in yeast [149]. These plasmids are called *shuttle* because they can propagate in both yeast and bacteria. The gene of interest is inserted into the episomal shuttle vector in vitro. Afterwards, bacterial—usually *E. coli*—and yeast cells are transformed with the newly assembled plasmid. *Escherichia coli* serves as a storage means, whereas yeast provides the machinery necessary for gene expression. However, *E. coli* can also be used, besides yeast and filamentous fungi, as an expression system for several kinds of LEs, as witnessed by the numerous works listed in Table 2.

**Table 2:** Source and examples of hosts that have been reported for expression of recombinant laccase within the last decade

Group	Source organism	Laccase type	Yeast hosts	References
Basidiomycota	Basidiomycete PM1 strain CECT 2971	PM1^g.e^	*Pichia pastoris*	[150, 151]
Basidiomycota	*Cerrena* sp. strain HYB07	Lac1–8	*P. pastoris*	[152]
Basidiomycota	*Coriolopsis gallica*	LCC1	*Kluyveromyces lactis*	[153]
Basidiomycota	*Cerrena unicolor* BBP6	Laccase	*Saccharomyces cerevisiae*	[154]
Basidiomycota	*Coprinopsis cinerea* Okayama-7#130	CcLCC2	*P. pastoris*	[155]
Basidiomycota	*C. cinerea*	LCC5I	*P. pastoris*	[156]
Basidiomycota	*C. cinerea*	LAC3^g.e.^, LAC4^g.e^	*P. pastoris*	[157]
Basidiomycota	*Ganoderm*a sp. En3	LAC-En3-1	*P. pastoris*	[158, 159]
Basidiomycota	*Trametes versicolor*	LCC	*S. cerevisiae*	[160]
Basidiomycota	*Trametes* sp. AH28-2	LACA	*S. cerevisiae*	[161]
Basidiomycota	*Trametes* sp. Ha1	Laccase I^g.e^	*S. cerevisiae*	[162]
Basidiomycota	*Trametes* sp. 5930	Lac5930-1	*P. pastors*	[163]
Basidiomycota	*Trametes* sp. 48424	LAC48424-1	*P. pastoris*	[164]
Basidiomycota	*T. versicolor*	LCC1^g.e^	*Yarrowia lipolytica*	[165]
Basidiomycota	*T. versicolor*	LCCA, LCCB,	*P. pastoris*	[166, 167]
Basidiomycota	*Trametes* sp. 5930	LAC5930-1	*P. pastoris*	[168]
Basidiomycota	*T. versicolor*	Lac6c	*S. cerevisiae EBY100*	[169]
Basidiomycota	*Lenzites gibbosa*	LAC	*P. pastoris*	[164]
Basidiomycota	*Pleurotus ostreatus*	POXA1b^g.e^	*S. cerevisiae*	[169]
Basidiomycota	*P. pulmonarius (PpuLcc)*	PpuLcc	*P. pastoris GS115*	[170]
Basidiomycota	*Pycnoporus sanguineus*	LCC1	*P. pastoris*	[171]
Basidiomycota	*P. ostreatus*	Lac-2	*P. pastoris X33*	[172]
Ascomycota	*Botrytis aclada*	LAC	*P. pastoris*	[173]
Ascomycota	*Monilinia fructigena*	LCC2	*P. pastoris*	[174]
Ascomycota	*Y. lipolytica*	YILAC	*P. pastoris*	[175]
Bacteria	*Aeromonas hydrophila* WL-11	Laccase	*Escherichia coli*	[176]
Bacteria	*Bacillus pumilus* strain W3	CotA–laccase	*E. coli*	[177]
Bacteria	*B. subtilis*	Lcc	*E. coli*	[178]
Bacteria	*B. subtilis, B. pumilus* and *B. clausii*	LMCOs	*E. coli*	[179]
Bacteria	*B. amyloliquefaciens* TCCC 111,018	LAC (rLAC)	*E. coli*	[6]
Bacteria	*B. velezensis* TCCC 111,904	Laccase (rLac)	*E. coli*	[180]
Bacteria	*Klebsiella pneumoniae*	rLac	*E. coli*	[181]
Bacteria	*Streptomyces coelicolor* A3(2)	rSLAC	*P. pastoris*	[182]
Bacteria	*S. griseorubens* JSD-1	MCO	*E. coli* TransB(DE3)	[183]
Bacteria	*Thermus thermophilus* SG0.5JP17-16	LACTt	*P. pastoris*	[184]
Bacteria	*T. thermophilus* HJ6	TtSLAC	*E. coli*	[185]
Bacteria	*Pseudomonas* and *Bacillus* species	Laccase CapR	*E. coli*	[186]
Bacteria	*Paenibacillus glucanolyticus *SLM 1	LMCO	*E. coli*	[187]
Bacteria	*Pandoraea* sp. ISTKB	Laccase	*E. coli *DH5α	[188]
Archaea	Archaea	LccA	*Haloferax volcani*	[189]

## Conclusion

Ligninolytic enzymes produced by fungi have been extensively exploited in a number of industries due to their versatile application, which derives from their lignin-based compounds catalytic oxidation properties. The large variety of lignin modifying fungal species and related ligninolytic enzyme consortium described in this review have been used in the production of biofuels, antibiotics, and fermented products, as well as in bioremediation and biomedical application as biosensors. The essential prerequisite for a successful use of fungal ligninolytic enzymes is their production in large quantities. Over the last decade, combined synthetic biology and computational designs have yielded significant results in enhancing the synthesis of natural compounds in fungi. Application of CRISPR-Cas system has shown great efficiency in discovery, activation, and editing of fungal genes/BGCs, and it holds a potential headway for production or enhancement of ligninolytic enzymes in large-scale. In addition, many newly available algorithms have been used to optimize CRISPR-Cas systems for editing specific loci or estimating potential off-target effects if used in fungal genomes. Further research could be directed towards improving the production of ligninolytic secretomes (or new ligninolytic genes) with desired attributes such as thermal, catalytic, or substrate adaptability. Various bacteria such as *E. coli* and *Bacillus subtilis* and filamentous fungi such as *Aspergillus* and *Trichoderma* species have been used as synthetic hosts for the expression of some LEs from white rot fungi. Yeasts such as *S. cerevisiae, P. pastoris, K. lactis*, and *Y. lipolytica* have been identified with good minicellulosomes, thus being good candidates for synthetic production of ligninolytic secretomes. Several studies have demonstrated heterologous expression in yeasts of ligninolytic enzymes from higher fungi. These re-engineered yeasts show adequate enzyme secretion with high tither values that do not affect negatively their cellular physiology. Yeast transformants in most cases showed better tolerance to oxidative stress, after the experiment, and ability to produce recombinant LEs in multiple folds, having better specification, quality, and reactivity as compared to the wild type. In addition, recombinant LEs can be easily characterized with molecular structure and studied for iron trafficking/hierarchy distribution in the cell.

## Supplementary Information

**Additional file 1: Table S1.** Fungal ligninolytic enzymes that are involved in the degradation of lignin, pesticides, drugs or hydrocarbons and their mediators. **Table S2. **Fungal Ligninolytic enzymes and their reactions in mediator containing media. **Table S3.** Example of some computation tools that can be used for pathway construction in fungi.

## Abbreviations

AAO: Aryl-alcohol oxidase
ABTS: 1-Hydroxybenzotriazole and 2,2-azinobis 3-ethylbenz-thiazoline-6-sulfonate
antiSMASH: Antibiotics and secondary metabolites analysis shell
AA: Auxiliary activity
BGCs: Biosynthetic gene clusters
BPs: Biosynthetic pathways
BQR: Benzoquinone reductase
CAD: Computer-aided design
CASSIS: Cluster assignment by islands of sites
CASTing: Combinatorial active site saturation testing
CAZy: Carbohydrate active enzymes
CB: Combinatorial biosynthesis
CDH: Cellobiose hydrogenase
CE: Chimeric enzymes
CDS: Coding sequence
CPO: Chloroperoxidase
CRISPR-Cas: Clustered Regularly Interspaced Short Palindromic Repeats and CRISPR-associated protein
CU: Codon usage
DE: Directed evolution
DyP: Dye-decolorizing peroxidase
ECC: Enzyme Commission classification
Eps: Enzymatic pathways
ER: Enzyme resurrection
GAO: Galactose oxidase
GLOX: Glyoxal oxidase
GMMs: Genome-scale metabolic models
GOX: Glucose oxidase
Hex: Heterologous expression
HTPs: Heme thiolate peroxidases
LB: Lignocellulose biorefinery
LCC: Laccase
LDA: Lignin-degrading auxiliary enzymes
LEs: Ligninolytic enzymes
LEC: Ligninolytic enzyme consortium
LiP: Lignin peroxidase
LMCO: Laccase-like multicopper oxidase
LMEs: Lignin-modifying enzymes
MF: Metabolic flux
MIBiG: Minimum Information about Biosynthetic Gene cluster
MnP: Manganese peroxidase
MOX: Methanol oxidase
MIDDAS-M: Motif-independent de novo detection algorithm for secondary metabolite biosynthetic gene clusters
NPs: Natural products
P2O: Pyranose 2-oxidase
RiPPs: Ribosomally synthesized and post-translationally modified peptides
RBS: Ribosome binding site
SBP: Silent biosynthetic pathways
SMIPS: Secondary metabolite by inter-pro-scan
SMURF: Secondary metabolite unique regions finder
TLS: The ligninolytic secretome
TALENs: Transcription Activator-like Effector Nucleases
UPOs: Nonspecific peroxygenases
VAO: Vanillyl-alcohol oxidase
Vp: Versatile peroxidase
WRF: White-rot fungi
