# Supplementary material for: Genome-based engineering of ligninolytic enzymes in fungi
Source: Microb Cell Fact. 2021 Jan 21;20:20. doi: 10.1186/s12934-021-01510-9 (PMC7819241; doi:10.1186/s12934-021-01510-9)
Supplement: Supplementary file 1 — Additional file 1: Table S1. Fungal ligninolytic enzymes that are involved in the degradation of lignin, pesticides, drugs or hydrocarbons and their mediators. Table S2. Fungal Ligninolytic enzymes and their reactions in mediator containing media. Table S3. Example of some computation tools that can be used for pathway construction in fungi. [file 12934_2021_1510_MOESM1_ESM.docx]

**Genome-based engineering of ligninolytic enzymes in fungi**

Michael Dare Asemoloye^1^, Mario Andrea Marchisio^1*^, Vijai Kumar Gupta^2^, Lorenzo Pecoraro^1*^

*^1^School of Pharmaceutical Science and Technology, Tianjin University, 92 Weijin Road, Nankai District, 300072, Tianjin, China.*

*^2^Biorefining and Advanced Materials Research Center, Scotland’s Rural College (SRUC), Kings Buildings, West Mains Road, Edinburgh, EH9 3JG, UK.*

*Corresponding authors: Mario Andrea Marchisio (telephone: +86 18526429643, e-mail: [mamarchisio@yahoo.com](mailto:mamarchisio@yahoo.com); [mario@tju.edu.cn](mailto:mario@tju.edu.cn)), Lorenzo Pecoraro (telephone: +86 18520824550, e-mail: [lorenzo.pecoraro@gmail.com](mailto:lorenzo.pecoraro@gmail.com); [lorenzo.pecoraro@tju.edu.cn](mailto:lorenzo.pecoraro@tju.edu.cn)) School of Pharmaceutical Science and Technology, Tianjin University, 92 Weijin Road, Nankai District, Tianjin, 300072, China.

**Table S1:** Fungal ligninolytic enzymes that are involved in the degradation of lignin, pesticides, drugs or hydrocarbons and their mediators

| **Fungal Enzyme** | **Cofactor or Substrate** |
| --- | --- |
| Lignin peroxidase, LiP | H_2_O_2_, veratryl alcohol |
| Manganese peroxidase, MnP | H_2_O_2_, Mn, organic acid as chelator, thiols, unsaturated lipids |
| Laccase, Lacc | O_2_; mediators, for example, hydroxybenzotriazole or ABTS |
| Versatile peroxidase (VP) | Reactive Black 5 |
| Glyoxal oxidase (GLX) | Glyoxal, methyl glyoxal |
| Aryl-alcohol oxidase (AAO) | Aromatic alcohols (anisyl, veratryl alcohol) |
| Dye-decolorizing peroxidase (DyP) | Reactive Black 5 |
| Chloroperoxidase (CPO) | Amino alcohol |
| Unspecific peroxygenase (UPO) | Aryl-alcohol |
| Cellobiose hydrogenase (CDH) | Ferrocene derivatives |
| Glucose oxidase (GOX) | Ferrocene derivatives |
| Methanol oxidase (MOX) | Ferrocene–methanol (12‐fold) and nitrosoaniline (8‐fold) |
| Pyranose 2-oxidase (P2O) | Ferrocene carboxylic acid; 2-ketoaldoses |
| Vanillyl-alcohol oxidase (VAO) | Vanillin, coniferyl alcohol and chiral aryl alcohols |
| Galactose oxidase (GAO) | Galactose and N-acetylgalactosamine |
| Benzoquinone reductase (BQR) | Quinone |

ABTS = 2,2′-Azino-bis(3-ethylbenzothiazoline-6-sulfonic acid)

**Table S2:** Fungal Ligninolytic enzymes and their reactions in mediator containing media

| **Enzyme** | **EC no.** | **Reaction** |
| --- | --- | --- |
| Lignin peroxidase (LiP) | 1.11.1.14 | (3,4-Dimethoxyphenyl)methanol + H_2_O_2_ ⇒ 3,4-di-methoxybenzaldehyde + 2 H_2_O |
| Manganese peroxidase (MnP) | 1.11.1.13 | 2 Mn(II) + 2 H^+^ + H2O2 ⇒ 2 Mn(III) + 2 H_2_O |
| Versatile peroxidase (VP) | 1.11.1.16 | (1) Reactive Black 5 + H_2_O_2_ ⇒ oxidized Reactive Black 5 + 2 H_2_O  (2) Donor + H_2_O_2_ = oxidized donor + 2 H_2_O |
| Laccase | 1.10.3.2 | 4 Benzenediol + O_2_ ⇒ 4 benzosemiquinone + 2 H_2_O |
| Dye-decolorizing peroxidase (DyP) | 1.11.1.19 | Reactive Blue 5 + 2 H_2_O_2_ ⇒ phthalate + 2,2′-disulfonyl azobenzene + 3-[(4-amino-6-chloro-1,3,5-triazin-2-yl)amino] benzenesulfonate + 2 H_2_O |
| Chloroperoxidase (CPO) | 1.11.1.10 | RH + chloride + H_2_O_2_ ⇒ RCl + 2 H_2_O |
| Unspecific peroxygenase (UPO) | 1.11.2.1 | RH + H_2_O_2_ ⇒ ROH + H_2_O |
| Cellobiose hydrogenase (CDH) | 1.1.99.18 | Cellobiose + acceptor ⇒ cellobiono-1,5-lactone + reduced acceptor |
| Aryl-alcohol oxidase (AAO) | 1.1.3.7 | Aromatic primary alcohol + O_2_ ⇒ aromatic aldehyde + H_2_O_2_ |
| Glucose oxidase (GOX) | 1.1.3.4 | β-D-Glucose + O_2_ ⇒ D-glucono-1,5-lactone + H_2_O_2_ |
| Methanol oxidase (MOX) | 1.1.3.13 | A primary alcohol + O_2_ ⇒ an aldehyde + H_2_O_2_ |
| Pyranose 2-oxidase (P2O) | EC1.1.3.10 | D-Glucose + O_2_ ⇒ 2-dehydro-D-glucose + H_2_O_2_ |
| Vanillyl-alcohol oxidase (VAO) | 1.1.3.38 | Vanillyl alcohol + O_2_ ⇒ vanillin + H_2_O_2_ |
| Glyoxal oxidase (GLX) | 1.1.3.– | RCHO + O_2_ + H_2_O ⇒ RCOOH + H_2_O_2_ |
| Galactose oxidase (GAO) | 1.1.3.9 | D-Galactose + O_2_ ⇒ D-galacto-hexodialdose + H_2_O_2_ |
| Benzoquinone reductase (BQR) | 1.6.5.6 | NADPH + H^+^ + p-benzoquinone ⇒ NADP+ + hydroquinone |

**Table S3:** Example of some computation tools that can be used for pathway construction in fungi

| **Category** | **Name** | **Database** | **Search algorithm** | **Supplementary Reference** |
| --- | --- | --- | --- | --- |
| Graph-based | ReTrace | KEGG | Heuristic search | [1] |
|  | PathComp | KEGG | Depth-first search (DFS) | [2] |
|  | MetaRoute | KEGG | Eppstein's k-shortest path [3] | |
|  | Pathway Hunter Tool | KEGG | BFS with HOHL | [4] |
|  | FMM | KEGG | BFS | [5] |
|  | RouteSearch | MetaCyc | Branch and Bound | [6] |
|  | MRE | KEGG | Yen's loopless k-shortest path | [7] |
|  | CMPF | KEGG, RPAIR | Bounded depth path enumeration | [8] |
|  | NeAT | MetaCyc | Takahashie Matsuyama, Pairwise K-shortest paths, and k Walks | [9] |
|  | LPAT/BPAT | KEGG | BPAT-M Search | [10] |
|  | Rahnuma | KEGG | DFS | [11] |
|  | Metabolic Tinker | CHEBI, Rhea | Heuristic search | [12] |
|  | FogLight | KEGG, MetaCyc | Brute-force search | [13] |
|  | MRSD | KEGG | Eppstein's k-shortest path [14] | |
|  | DESHARKY | KEGG | Monte Carlo | [15] |
| Stoichiometry based | optStoic | KEGG, MetRxn | MILP | [16] |
|  | PathTracer | BIGG, Ijo1366 | MILP | [17] |
|  | CFP | BIGG | MILP | [18] |
|  | METATOOL 5.0/kshortest FFM | BIGG, iAF1260 | MILP | [19, 20] |
|  | OptStrain | KEGG | MILP | [21] |
| Retrosynthesis based | Simpheny | BIGG | Retrosynthetic enumeration | [22] |
|  | GEM-Path | BIGG, iJO1366 | Retrosyntheticenumeration | [23] |
|  | XTMS/ RetroPath/ RetroPath 2.0 | MetaCyc, BioCyc | Retrosynthetic enumeration and MILP | [24-26] |
|  | BNICE | KEGG, ATLAS | Retrosynthetic enumeration | [27] |
|  | UM-PPS | UM-BBD | Retrosynthetic enumeration | [28] |
|  | PathPred | KEGG, RPAIR | Retrosynthetic enumeration | [29] |
|  | Route Designer | MOS, Beilste | Retrosynthetic enumeration | [30] |
|  | SimIndex/ SimZyme | BRENDA | ByerseWaterman type pathway search | [31] |
|  | Method by Cho et al. | KEGG | Retrosynthetic enumeration | [32] |

*Table adapted from Wang et al. (2017)*

**Further information**

Active Site Classification (ASC): http://asc.informatik.uni-tuebingen.de

antiSMASH: http://antismash.secondarymetabolites.org

Asmparts: http://soft.synth-bio.org/asmparts.html

Biojade: http://web.mit.edu/jagoler/www/biojade/

BioMet Toolbox: http://www.sysbio.se/BioMet/

BioNetCAD: http://www.sysdiag.cnrs.fr/BioNetCAD/

CarbonSearch: http://www.kavrakilab.org/atommetanet

CellDesigner: http://www.celldesigner.org

CloneQC: http://cloneqc.thruhere.net

Clotho: http://www.clothocad.org

COBRA Toolbox: http://opencobra.sourceforge.net

CycSim: http://www.genoscope.cns.fr/cycsim/

DESHARKY: http://soft.synth-bio.org/desharky.html

DNAWorks: http://helixweb.nih.gov/dnaworks/

From Metabolite to Metabolite (FMM): http://fmm.mbc.nctu. edu.tw

Gene Composer: http://www.genecomposer.net

Gene Designer 2.0: https://www.dna20.com/genedesigner2/

GeneDesign: http://www.genedesign.org

GenoCAD: http://www.genocad.org

GLAMM: http://glamm.lbl.gov

Integrated Microbial Genomes (IMG): http://img.jgi.doe.gov

iPATH2: http://pathways.embl.de

KEGG: http://www.genome.jp/kegg/

MultiGeneBlast: http://multigeneblast.sourceforge.net/

Optimizer: http://genomes.urv.cat/OPTIMIZER/

RBS Calculator: https://salis.psu.edu/software/

RBSDesigner: http://rbs.kaist.ac.kr

Registry of Standard Biological Parts: http://partsregistry.org

RetroPath: http://www.issb.genopole.fr/~faulon/retropath. php

Standard Biological Parts knowledgebase: http://sbpkb. sbolstandard.org

SurreyFBA: http://sysbio3.fhms.surrey.ac.uk/SurreyFBA.zip

SynBioSS: http://synbioss.sourceforge.net

TinkerCell: http://www.tinkercell.com

WebGEC: http://lepton.research.microsoft.com/webgec/

**Supplementary Reference**

1. Pitkanen E, Jouhten P, Rousu J. Inferring branching pathways in genomescale metabolic networks. BMC Syst. Biol. 2009;3:103.
2. Goto S, Bono H, Ogata H, et al. Organizing and computing metabolic pathway data in terms of binary relations. *Pac Symp Biocomput*. 1997;175-186.
3. Blum T, Kohlbacher O. MetaRoute: fast search for relevant metabolic routes for interactive network navigation and visualization. Bioinform. 2008;24(18):2108e9.
4. Rahman SA, Advani P, Schunk R. et al. Metabolic pathway analysis web service (Pathway Hunter Tool at CUBIC). Bioinform. 2005;21(7):1189e93.
5. Chou CH, Chang W, Chiu C et al. FMM: a web server for metabolic pathway reconstruction and comparative analysis. Nucleic Acids Res. 2009;37:W129e34.
6. Latendresse M, Krummenacker M, Karp PD. Optimal metabolic route search based on atom mappings. Bioinform. 2014;30(14):2043e50.
7. Kuwahara H, Alazmi M, Cui X, Gao X. MRE: a web tool to suggest foreign enzymes for the biosynthesis pathway design with competing endogenous reactions in mind. Nucleic Acids Res. 2016;44(W1):W217e25. <https://doi.org/10.1093/nar/gkw342>
8. Lim K, Wong L. CMPF: class-switching minimized pathfinding in metabolic networks. BMC Bioinforma. 2012;13(Suppl 17):S17.
9. Faust K, Dupont P, Callut J, van Helden J: Pathway discovery in metabolic networks by subgraph extraction. Bioinformatics 2010;26:1211-1218.
10. Heath AP, Bennett GN, Kavraki LE. An algorithm for efficient identification of branched metabolic pathways. J. Comput. Biol. 2011;18(11):1575e97.
11. Mithani A, Preston GM, Hein J. Rahnuma: hypergraph-based tool for metabolic pathway prediction and network comparison. Bioinform. 2009;25(14):1831e2.
12. McClymont K, Soyer OS. Metabolic tinker: an online tool for guiding the design of synthetic metabolic pathways. Nucleic Acids Res 2013;41(11), e113.
13. Khosraviani M, Saheb ZM, Bidkhori G. FogLight: an efficient matrixbased approach to construct metabolic pathways by search space reduction. Bioinform. 2016;32(3):398e408.
14. Xia D, Zheng H, Liu Z. et al. MRSD: a web server for metabolic route search and design. Bioinform. 2011;27(11):1581e2. <https://doi.org/10.1093/bioinformatics/btr160>
15. Rodrigo G, Carrera J, Prather JK, Jaramillo A. DESHARKY: automatic design of metabolic pathways for optimal cell growth. Bioinform. 2008;24(21):2554e6.
16. Chowdhury A, Maranas CD. Designing overall stoichiometric conversions and intervening metabolic reactions. Sci. Rep. 2015;5.
17. Tervo CJ, Reed JL. MapMaker and PathTracer for tracking carbon in genomescale metabolic models. Biotechnol. J. 2016;11(5):648e61.
18. Pey J, Prada J, Beasley J.E. et al. Path finding methods accounting for stoichiometry in metabolic networks. Genome Biol. 2011;12(5):R49. https://doi.org/10.1186/gb-2011-12-5-r49
19. von Kamp A, Schuster S, Metatool S. 5.0: fast and flexible elementary modes analysis. Bioinform. 2006;22(15):1930e1.
20. de Figueiredo LF, Podhorski A, Rubio A. et al. Computing the shortest elementary flux modes in genome-scale metabolic networks. Bioinform. 2009;25(23);3158e65. <https://doi.org/10.1093/bioinformatics/btp564>
21. Pharkya P, Burgard AP, Maranas CD. OptStrain: a computational framework for redesign of microbial production systems. Genome Res. 2004;14(11):2367e76.
22. Yim H. Haselbeck R, Niu W. et al. Metabolic engineering of Escherichia coli for direct production of 1,4-butanediol. Nat. Chem. Biol. 2011;7(7):445e52. https://doi.org/10.1038/nchembio.580
23. Campodonico MA, Andrews BA, Asenjo J. et al. Generation of an atlas for commodity chemical production in Escherichia coli and a novel pathway prediction algorithm, GEM-Path. Metab. Eng. 2014;25:140e58. [https://doi.org/10.1016/j.ymben.2014.07.009](https://doi.org/10.1016/j.ymben.2014.07.009)
24. Carbonell P, Parutto P, Herisson SJ, Pandit SB, Faulon J. XTMS: pathway design in an eXTended metabolic space. Nucleic Acids Res. 2014;42;W389e94. <https://doi.org/10.1093/nar/gku362>
25. Carbonell P, Planson AG, Fichera D, Faulon JL. A retrosynthetic biology approach to metabolic pathway design for therapeutic production. BMC Syst. Biol. 2011;5:122. doi:10.1186/1752-0509-5-122
26. Delepine B. et al. RetroPath2.0: a retrosynthesis workflow for metabolic engineers. bioRxiv; 2017;141721.
27. Hatzimanikatis V, Li C, Justin A. et al. Exploring the diversity of complex metabolic networks. Bioinform. 2005;21(8):1603e9. <https://doi.org/10.1093/bioinformatics/bti213>
28. Gao J, Ellis LB, Wackett LP. The university of Minnesota pathway prediction system: multi-level prediction and visualization. Nucleic Acids Res. 2011;39:W406e11.
29. Moriya Y, Shigemizu D, Hattori M. et al. PathPred: an enzyme-catalyzed metabolic pathway prediction server. Nucleic Acids Res. 2010;38:W138e43.
30. Law J. Zsoldos Z, Simon A. et al. Route Designer: a retrosynthetic analysis tool utilizing automated retrosynthetic rule generation. J. Chem. Inf. Model. 2009;249(3):593e602.
31. Pertusi DA. Stine AE, Broadbelt LJ, Tyo KE. Efficient searching and annotation of metabolic networks using chemical similarity. Bioinform. 2015;31(7):1016e24. https://doi.org/10.1093/bioinformatics/btu760.
32. Cho A. Yun, H., Park, J.H. et al. Prediction of novel synthetic pathways for the production of desired chemicals. BMC Syst. Biol. 2010;4:35. https://doi.org/10.1186/1752-0509-4-35
